# Sphingolipid and Ceramide Homeostasis: Potential Therapeutic Targets

**DOI:** 10.1155/2012/248135

**Published:** 2012-02-09

**Authors:** Simon A. Young, John G. Mina, Paul W. Denny, Terry K. Smith

**Affiliations:** ^1^School of Biology and Chemistry, Biomedical Sciences Research Complex, University of St Andrews, North Haugh, KY16 9ST, UK; ^2^Biophysical Sciences Institute, School of Biological and Biomedical Sciences and Department of Chemistry, University of Durham University Science Laboratories, South Road, Durham DH1 3LE, UK; ^3^School of Medicine and Health, Durham University, Queen's Campus, Stockton-on-Tees TS17 6BH, UK

## Abstract

Sphingolipids are ubiquitous in eukaryotic cells where they have been attributed a plethora of functions from the formation of structural domains to polarized cellular trafficking and signal transduction. Recent research has identified and characterised many of the key enzymes involved in sphingolipid metabolism and this has led to a heightened interest in the possibility of targeting these processes for therapies against cancers, Alzheimer's disease, and numerous important human pathogens. In this paper we outline the major pathways in eukaryotic sphingolipid metabolism and discuss these in relation to disease and therapy for both chronic and infectious conditions.

## 1. Introduction

**Figure 1:**
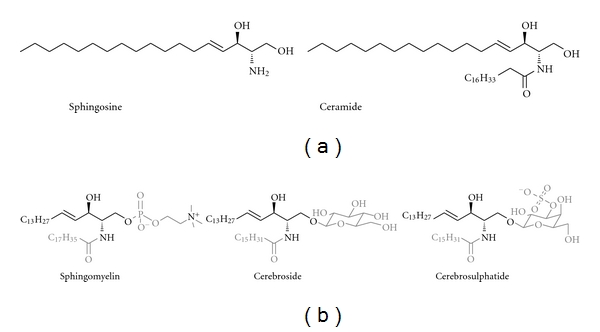
(a) The chemical structures of sphingosine and C_18_-ceramide; (b) the lipids isolated by Thudichum.

Sphingolipids are a class of natural products that were first characterised by the German-born chemist and clinician Johann L. W. Thudichum in 1884. They consist of an sphingoid base backbone, for example sphingosine, that can be N-acylated with fatty acids forming ceramides. To these lipid anchors is attached a variety of charged, neutral, phosphorylated and/or glycosylated moieties forming complex sphingolipids, for example phosphorylcholine to make the most abundant mammalian sphingolipid, sphingomyelin. These moieties result in both polar and nonpolar regions giving the molecules an amphipathic character which accounts for their tendency to aggregate into membranous structures. Furthermore, the divergence encountered in their chemical structures allows them to play distinctive roles within cellular metabolism (Figure 1) [1, 2].

Sphingolipids are ubiquitous and essential structural components of eukaryotic membranes [3] as well as some prokaryotic organisms and viruses [2]. They are found predominantly in the outer leaflet of the plasma membrane [4], the lumen of intracellular organelles [5], and lipoproteins [2]. Sphingolipids (most notably ceramide) are also bioactive signalling molecules that control a plethora of cellular events including signal transduction, cell growth, differentiation, and apoptosis [6–10]. In addition, their role in protein kinase C regulation has more recently been elucidated [11]. It is noteworthy that some sphingolipid metabolites can exhibit both structural and signalling functionalities. For example, glycosphingolipids have been reported to be involved in cellular recognition complexes, for example, blood group antigens, cell adhesion, and the regulation of cell growth [4].

Over the last decade, there has been an exponential increase in the study of sphingolipids. However, the investigation and deciphering of the functions of each specific sphingolipid remains challenging due to the complexity in sphingolipid metabolic interconnection, their varied biophysical properties (neutral or charged), the hydrophobic nature of the enzymes involved, and the presence of multiple pathways that can operate in parallel [12]. The interaction of sphingolipid biosynthesis with other cellular metabolic pathways, for example glycerolipid metabolism, introduces another layer of complexity and the cellular role of an individual sphingolipid could be defined as a multidimensional in terms of subcellular localisation, regulation and mechanism of action(s) [12].

Whilst the scientific literature has been enriched by articles focused on structural diversity [13–18] and cellular metabolism [2, 8–10, 12, 19–21], this paper focuses on the key enzymes involved in the regulation of ceramide, a central sphingolipid and a key bioactive molecule [12]. To this end we discuss the roles of these enzymes in the regulation of biosynthesis, and in the recycling, salvage, and degradation of complex sphingolipids, in mammalian, fungal and protozoan systems. In addition, we relate these observations to disease and potential therapies.

## 2. Sphingolipid Metabolism

**Figure 2:**
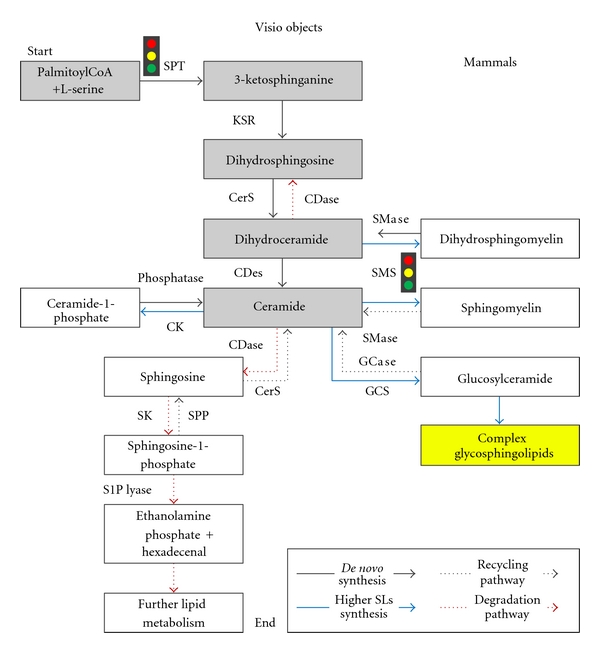
A simplified diagram of sphingolipid metabolism in mammals. The key regulatory synthetic steps are indicated by the “traffic light” symbols. SPT: Serine Palmitoyltransferase; 3-KSR: 3-Ketosphingosine Reductase; CerS: Ceramide Synthase; CDase: Ceramidase; CDes: Ceramide Desaturase; SMS: Sphingomyelin Synthase; SMase: Sphingomyelinase; CK: Ceramide Kinase; GCS: Glucosylceramide Synthase; GCase: Glycosidases; SK: Sphingosine Kinase; SPP: Sphingosine-1-Phosphate Phosphatase; S1P: Sphingosine-1-Phosphate.

Sphingolipid metabolism is a critical cell process [22] constituting a highly complex network of interconnected pathways, with ceramide (and to a lesser degree dihydroceramide) occupying a central position in both biosynthesis and catabolism. Therefore, this simple but highly bioactive sphingolipid represents a metabolic hub [12]. In terms of ceramide, the routes of formation can be grouped into either *de novo* synthesis; or recycling, salvage, and degradation (Figure 2). Sphingolipid metabolism has been extensively studied in mammalian and fungal systems, where many of the enzymes involved have been identified and characterised. Consequently, the mammalian pathways will be used as the reference model in the following discussion.

### 2.1. *De Novo* Synthesis

The first step in the *de novo* biosynthesis of sphingolipids is the condensation of serine and palmitoyl CoA, a reaction catalysed by the normally rate-limiting serine palmitoyltransferase (SPT, EC 2.3.1.50) to produce 3-ketodihydrosphingosine [23]. SPTs are members of the pyridoxal 5′-phosphate-dependent *α*-oxoamine synthase family who share a conserved motif (T[FL][GTS]**K**[SAG][FLV]G) around the PLP-binding lysine (in bold). The mammalian SPTs [23] (and those of other eukaryotes [24, 25]) are membrane bound in the endoplasmic reticulum as a heterodimer of subunits LCB1 and LCB2 (~53 and ~63 kDa); these are both type I integral membrane proteins sharing ~20% identity. The bacterial SPT is ~30% identical to both mammalian LCB1 and LCB2 at the amino acid level and has the conserved lysine residue in the PLP-binding motif, however the soluble 45 kDa protein forms active homodimers [26]. Palmitoyl-CoA functions as the best substrate of mammalian SPT *in vitro*, while it is also the dominant acyl-CoA *in vivo*, and thus the sphingoid bases from mammalian cells are predominantly C16 [23]. In contrast, the enzyme from the bacteria *Sphingomonas wittichii* utilises stearoyl-CoA most efficiently [27]. A third subunit increasing enzyme activity has been identified in *Saccharomyces cerevisiae* [28] and more recently 2 nonhomologous but functionally related proteins have been characterised in a mammalian system [29]. Furthermore, these additional subunits confer distinct acyl-CoA substrate specificities to the mammalian SPT thus explaining the diversity of long chain bases found in mammals [30]. As the “gatekeeper” of sphingolipid biosynthesis, loss of SPT has a catastrophic effect on mammalian cell viability with a partial loss of SPT function seen in the inherited progressive disorder, Hereditary Sensory Neuropathy type I (HSN1) [23]. The molecular basis of this condition is discussed later in this paper.

**Figure 3:**
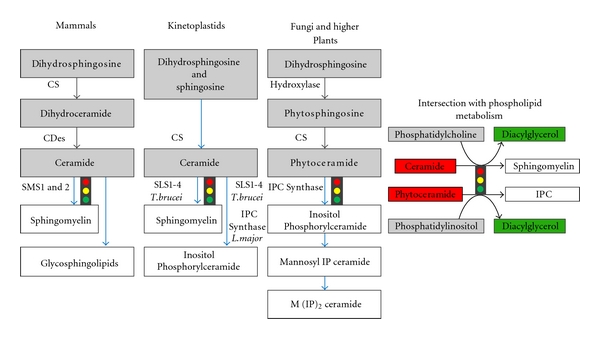
Divergence in the postceramide biosynthetic steps highlighting the conserved intersection with phospholipid metabolism as a regulator of the balance between promitogenic diacylglycerol (DAG) and proapoptotic ceramide. The key regulatory synthetic steps are indicated by the “traffic light” symbols. CS: Ceramide Synthase; CDes: Ceramide Desaturase; SMS: Sphingomyelin Synthase; SL: Sphingolipid; IPC: Inositol Phosphorylceramide; PC: Phosphatidylcholine.

Following sphinganine (dihydrosphingosine) formation, metabolic differences are encountered. Whilst in fungi and higher plants sphinganine is hydroxylated to phytosphingosine then acylated to produce phytoceramide, in animal cells sphinganine is acylated to dihydroceramide which is later desaturated to form ceramide [31]. Ceramide (or phytoceramide), a central sphingolipid, is then transported from the ER to the Golgi apparatus where further synthesis of complex sphingolipids takes place [7, 19, 20, 32]. Ceramide can be phosphorylated by ceramide kinase [33], glycosylated by glucosyl or galactosyl ceramide synthases [34], or acquire a variety of neutral or charged head groups to form various complex phosphosphingolipids depending on the host organism. For example, in animal cells ceramide is a substrate for sphingomyelin (SM) synthase to produce SM [35]. In contrast, fungi and higher plants utilise phytoceramide to produce inositol phosphorylceramide (IPC) as their principal phosphosphingolipid, a reaction catalysed by IPC synthase [36, 37]. In these organisms IPC is later glycosylated to produce more complex phosphosphingolipids, for example, mannose-IPC (MIPC), in yeast [38, 39]. Finally, the protozoa (exemplified by the Kinetoplastidae) represent a distinct third group in which ceramide [21] acquires a phosphorylinositol head group from phosphatidylinositol (PI) to produce IPC via IPC synthase [40] (Figure 3).

The synthesis of complex sphingolipids such as SM are key regulatory synthetic steps, as the rate of synthesis not only decreases the amount of ceramide, but also indirectly increases the total amount of ceramide-containing molecules that potentially could be degraded/catabolised to form ceramide.

Importantly, in such biosynthetic steps the evolutionarily divergent SM and IPC synthases are central in controlling the delicate balance of glycerolipids (PI/PC in and diacylglycerol-DAG out) on one hand, and sphingolipids (phytoceramide/ceramide in and IPC/SM out) on the other. Therefore, these enzymes have an important role as regulators of proapoptotic ceramide and promitogenic DAG [41]. In addition to a mitogenic role, DAG has also been attributed to play a role in several enzyme activation and regulatory functions [1, 2]. Notably, IPC synthase inhibitors are acutely fungicidal, with the accumulation of ceramide proposed to induce apoptosis [42]. Thus this enzyme represents an attractive target for antifungals and more recently this has been extended to the kinetoplastid protozoa [43, 44].

### 2.2. Recycling, Salvage, and Degradation

In addition to the *de novo* synthesis, the recycling, salvage, and degradation pathways modulate cellular levels of sphingolipids. These pathways operate in the direction of ceramide regeneration from complex sphingolipid reservoirs, for example glycosphingolipids (GSLs) and (SMs), through the action of specific hydrolases and phosphodiesterases.

Sphingolipid recycling can be categorised as either lysosomal or nonlysosomal degradation. In lysosomal degradation, catabolism of GSLs occurs as sugar residues are cleaved leading to the formation of glucosylceramide and galactosylceramide. In turn, specific *β*-glucosidases and galactosidases hydrolyse these lipids to form ceramide which can then be subsequently deacylated by an acid ceramidases to form sphingosine [12, 45], which can then be salvaged to form ceramide by reacylation. Defects in the function of these enzymes lead to a variety of lysosomal storage disorders such as Gaucher, Sandhoff, and Tay-Sachs diseases, resulting from the impairment of membrane degradation [46]. This will be discussed in more detail later.

Degradation of sphingolipids is a necessary part of maintaining lipid homeostasis, thus SM levels are maintained by catabolic action of sphingomyelinases (SMases), either neutral or acidic, releasing ceramide and the corresponding headgroup, phosphorylcholine in the case of SM. Acid sphingomyelinase (aSMase) was the first cloned human SMase and was initially assumed to be not much more than a housekeeping gene with a prominent role in the turnover of sphingomyelin in the lysosome. However, unusually aSMase has revealed itself to encode two unique enzymes through the differential trafficking of a single-protein precursor [47]. In addition to the commonly studied lysosomal aSMase, an alternative form is secreted extracellularly and may have a role in the nonlysosomal hydrolysis of SM both in the outer leaflet of the plasma membrane and in lipoproteins in the bloodstream [48]. These studies indicate that ceramide production by sphingolipid hydrolysis in different cellular or extracellular locations may provide different metabolic effects and biological impacts. Indeed, the nonlysosomal degradation of SM is catalysed by neutral and alkaline SMases in a variety of intracellular and extracellular locations. The least studied of these SMases, the alkaline SMase (Alk-SMase), is highly tissue specific with trypsin resistance and bile salt dependency and has a key role in the dietary acquisition of ceramide by digesting SM in the gut [49]. Notably, animal studies have shown Alk-SMase is specifically down regulated in colon cancer, while membrane SM accumulates. The supplementation of dietary SM can prevent the promotion of further colonic tumors [50].

SMases are commonly activated by growth factors, cytokines, chemotherapeutic agents, irradiation and nutrient removal [51], and though they can differ in their subcellular localisation and tissue specificity, all are thought to regulate the local ceramide concentration and any corresponding stress-induced responses [52]. Ceramide and associated metabolites, such as sphingosine-1-phosphate, are known to function as second messengers, stimulating various biological activities in mammalian cells, including the activation of protein-kinases and/or protein-phosphatases 2A [53]. Increased levels of ceramides can exert antiproliferation effects, induce apotosis, and play major roles in mitogenesis and endocytosis. There is a growing body of evidence that suggests Mg^2+^-dependent neutral sphingomyelinases (nSMases) are the major source for stress-induced ceramide production [51]. nSMases are ubiquitously expressed in mammalian cells, predominately membrane bound on the outer leaflet of the plasma membrane where most of the SM is located [52]. Other mammalian nSMases localise to the ER, where the predicted low abundance of SM has led to speculation that they may have additional lipid substrates such as lyso-platelet-activating factor [54]. In all cases of sphingolipid catabolism, the released ceramide can be either recycled into sphingolipid synthesis or degraded to sphingosine [12, 45]. The resultant sphingosine, produced from either pathway, is either recycled into sphingolipid biosynthesis or phosphorylated by a cytosolic sphingosine kinase (SK) yielding sphingosine-1-phosphate (S1P). S1P can itself be dephosphorylated back to sphingosine or irreversibly degraded by S1P lyase into the nonsphingolipid species ethanolamine phosphate and hexadecenal, representing a unique exit point from the sphingolipid metabolic pathway [12, 45]. In fact this is the mechanism by which the kinetoplastid *Leishmania* obtain ethanolamine [21].

Another kinetoplastid, *Trypanosoma brucei*, has shown that an ER nSMase directly involved in sphingolipid catabolism is essential because its formation of ceramide is required for post-Golgi sorting and deposition of the essential glycosylphosphatidylinositol-anchored variant surface glycoprotein on the cell surface [55]. Similarly, the *Leishmania* nSMase is essential for virulence and, whilst able to catabolise inositol phosphorylceramide (IPC), demonstrated greater activity with SM [56].

The corresponding yeast nSMase homologue (Isc1) is also capable of IPC catabolism, generating ceramide. During early growth Isc1p resides in the ER, but in late logarithmic growth it is found in the outer leaflet of the mitochondria, where the resulting ceramide formation plays a crucial role in the reprogramming of mitochondrial gene expression during the transition from anaerobic to aerobic metabolism, coupled with a change in carbon source, that is, glucose to ethanol [57, 58].

## 3. Ceramide Homeostasis

As discussed, ceramides are central intermediates of sphingolipid metabolism. In addition to forming the basis of complex sphingolipids, ceramide is a bioactive molecule that regulates a myriad of cellular pathways including apoptosis, cell senescence, the cell cycle, and differentiation [59]. In addition, this lipid species is involved in the cell response to stress challenge. Notably, several anticancer drugs, for example, etoposide and daunorubicin, have been found to function by elevating the level of cellular ceramide triggering apoptosis [60–63]. The apoptotic role of ceramide [64, 65] contrasts with that of the mitogenic agonist DAG. Whilst the former stimulates signal transduction pathways associated with cell death or growth inhibition, DAG activates the various isoforms of protein kinase C associated with cell growth and survival. Thus, ceramide and DAG generation may regulate cellular homeostasis by inducing death and growth, respectively. Given that ceramide and DAG are a substrate and a byproduct, respectively, of SM and IPC synthases, these enzymes are hypothesized to play a central role in homeostasis [9].

Recently, a human SM synthase-related protein has been shown to function as a ceramide sensor [66] with a crucial role in protecting cells against ceramide-induced cell death. Disruption of this sensor leads to ceramide accumulation in the endoplasmic reticulum and mitochondrial-mediated apoptosis. This process is suppressed by targeting a ceramidase to mitochondria indicating that transfer of ceramide from the endoplasmic reticulum to mitochondria, via an unknown mechanism, is a key step in committing cells to death. The presence of a mitochondrial ceramide synthase has also been reported and hypothesized to play a role in this apoptotic process [67]. Together, these findings provide mechanistic evidence for the proapoptotic accumulation of ceramide in the mitochondria and demonstrate that the regulation of ceramide homeostasis is a vital cellular function.

## 4. Defects in Sphingolipid Metabolism

Despite sphingolipids being minor components in some cells, their accumulation in certain cells and tissues forms the basis of many human diseases.

### 4.1. Sphingolipidoses

**Figure 4:**
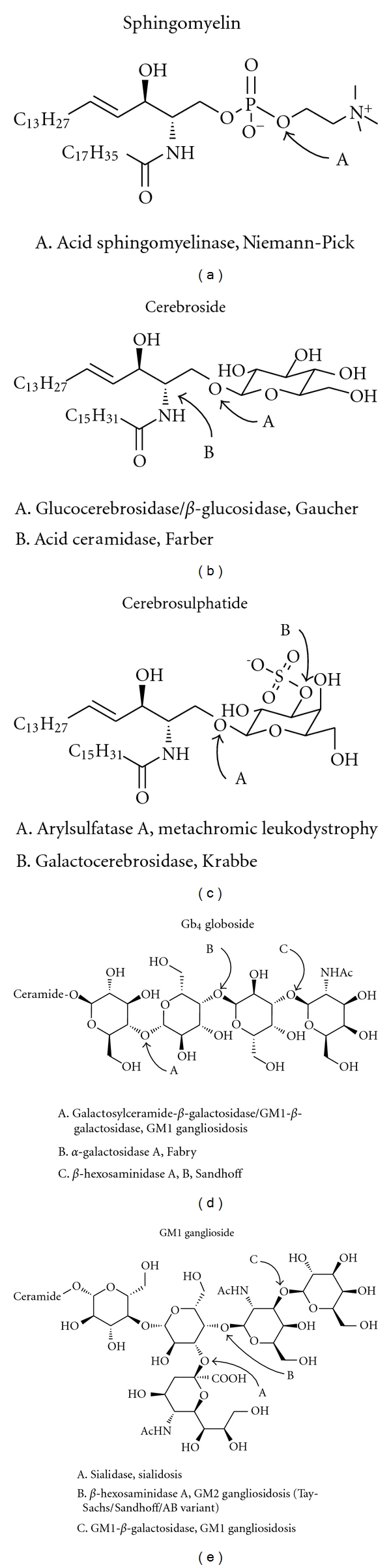
Defects in mammalian sphingolipid catabolism and their associated sphingolipidoses. The enzymes responsible are indicated at the glycosidic linkages on which they act.

Defects in sphingolipid catabolism, that is, lipid hydrolases, form the basis of a wide variety of human diseases. These diseases, collectively known as sphingolipidoses (Figure 4), belong to the lysosomal storage diseases and are inherited disorders characterised by accumulations in specific lipids in certain tissues and/or organs.

The most common is Gaucher disease, in which glucosylceramide accumulates due to a deficiency of glucosylceramide-*β*-glucosidase, causing changes in the specialised membrane microdomains termed lipid rafts. This in turn seems to impair lipid and protein sorting and consequently causes the pathology characterising this disorder. For example, lipid rafts are necessary for correct insulin signalling, and a perturbed lipid raft composition impairs insulin signalling leading to the insulin-resistance observed in patients with Gaucher disease [68, 69].

Fabry disease is an X-chromosomal-linked inherited deficiency of lysosomal *α*-galactosidase A, causing deposition of globotriaosylceramide in the lysosomes of endothelial, perithelial and smooth-muscle cells of blood vessels. This leads to renal, cardiac and/or cerebral complications and, most commonly, death before the age of 50 [70, 71].

Tay-Sachs disease or GM2-gangliosidosis comes in various forms, the most extreme, infantile form being caused by defects in *β*-hexosaminidase A and has a high heterozygote frequency (1 : 27) among Ashkenazi Jews. This condition leads to death between the second and fourth years of life [72, 73].

Sandhoff disease is characterized by storage of negatively charged glycolipids and elevation of uncharged glycolipids. This disease has various clinical forms, infantile, juvenile, and adult, all with varying pathological manifestations, including a chronic variant similar to Tay-Sachs disease [74, 75].

The inherited Niemann-Pick disease (types A and B) is characterised by a deficiency in the lysosomal acidic SMase, causing an accumulation not only of sphingomyelin but also of glycosphingolipids, sphingosine and others in multilamellar storage bodies [76].

There are many other related and associated genetic diseases, Metachromatic Leukodystrophy caused by a deficiency of arylsulfatase A, Krabbe disease or globoid cell leukodystrophy caused by a deficiency of galactosylceramide-*β*-galactosidase and Farber disease caused by a deficiency of lysosomal acid ceramidase causing storage of excess ceramide in the lysosomes. For further details of these and other sphingolipid metabolic diseases, refer to an excellent review by Kolter and Sandhoff [77].

### 4.2. Hereditary Sensory Neuropathy

Clinical disorders are also associated with alterations in SPT activity, although a complete lack of SPT activity is predicted to be embryonically lethal. The inherited disease hereditary sensory neuropathy type I (HSN1) is a progressive degeneration of lower limb sensory and autonomic neurons and has been associated with mutations in the human *LCB1* gene [78, 79]. LCB1 (SPTLC1) mutations confer dominant negative effects on SPT, thus substantially reducing SPT activity and hence the rate of *de novo* synthesis of sphingolipids [80]. Recently, point mutations have been found in the catalytic SPT subunit (LCB2 or SPTLC2) in patients suffering from HSN1. These were confirmed to affect SPT activity using an *in vitro* system. No mutations were observed in the third (SPTLC3) subunit in these patients [81].

Point mutations in the SPT complex also affect SPT activity in terms of substrate specificity and alanine can be used instead of serine in the condensation with palmitoyl-CoA, resulting in the formation and accumulation of 1-deoxy-sphinganine in the serum of these patients [82].

### 4.3. Alzheimer's Disease

A relatively recent discovery was the highly altered sphingolipid metabolism in brain cells in Alzheimer's disease [83]. The latest study has highlighted the key role of an nSMase in the disease by promoting the damaging effects of fibrillar amyloid-*β* 1–42 peptide-activated astroglia through ceramide production and thus apoptosis in neuronal cells [84].

In addition, SPT has been shown to be downregulated by the amyloid precursor protein [85]. This novel physiological function of the amyloid precursor protein suggests that SPT and sphingolipid metabolism is involved in Alzheimer's disease pathology.

### 4.4. Other Diseases

Obesity and its established association with insulin resistance, type 2 diabetes, and cardiovascular disease are directly and/or indirectly involved in the overaccumulation of long-chain fatty acids. The resulting surplus to the storage capacity of adipose tissue results in deposition in nonadipose tissues, such as the liver, muscle, heart, and pancreatic islets. This leads to deleterious effects, not only as atherosclerosis, but excess lipids are also forced into alternative nonoxidative pathways resulting in the formation of reactive lipid moieties, such as sphingolipids, that promote metabolically relevant cellular dysfunction (lipotoxicity) and programmed cell death (lipoapoptosis) [86–88].

## 5. Sphingolipid Biosynthesis: An Attractive Drug Target

Due to the complexities of sphingolipid metabolism and associated defects in a variety of tissue types and cell compartments, there is a significant challenge in the understanding, diagnosis, and treatment of genetic diseases such as those discussed above. However, clinical manipulation sphingolipid metabolism will prove key in the treatment of these conditions; in addition it is becoming clear that by inducing the accumulation of proapoptotic ceramide, therapies for cancer and infectious disease may be developed.

### 5.1. Sphingolipidoses

The general strategy to treating the inherited human diseases involving sphingolipid metabolism, the sphingolipidoses is the restoration of the defective lysosomal degradation. These include enzyme replacement, heterologous bone marrow transplantation as a form of cell-mediated therapy, gene therapy and the use of chemical chaperones for enzyme-enhancement therapy. An alternative strategy is the reduction of substrate influx into the lysosomes using substrate reduction (substrate deprivation) therapy ([77] and references therein).

### 5.2. Cancer

The roles of ceramide in diverse cellular responses to stress, particularly apoptosis, has been discussed above and as mentioned elevated ceramide often result from treatment with anticancer drugs and also irradiation. Therefore the manipulation of sphingolipid biosynthesis and homeostasis to elevate ceramide levels and induce programmed cell death is a viable strategy for anticancer therapies [89]. Conversely, dysregulation of ceramide metabolism affects the cellular response to chemotherapy or other anticancer regimens by rendering the cells more resistant to killing; in these cases therapeutic manipulation of ceramide metabolism could overcome this resistance [90, 91]. Further developments in the manipulation of sphingolipid metabolism as an anticancer strategy will undoubtedly follow with the breakthrough discovery that FTY720, a water-soluble sphingosine analogue effective in many cancer models which acts by downregulating nutrient transporter proteins in cancer cells at least partially via ceramide generation [92]. The resulting starvation induces a homeostatic autophagy selectively in cancer cells sensitive to nutrient limitation, while normal cells have the ability to adapt and survive by becoming quiescent. Notably AAL-149, an FTY720 analogue, similarly kills patient-derived leukaemic cells, but not cells of healthy donors, without the dose-limiting toxicity of FTY720. Thus, by targeting the sphingolipid metabolism of cancer cells rather than any specific oncogenic defect, such compounds should have potent activity against a range of tumours, particularly if applied in combination with inhibitors of autophagy.

### 5.3. Pathogens

The essential functions of sphingolipids, coupled with the divergence of the biosynthetic pathway between mammals and eukaryotic pathogens have resulted in the investigation of the biosynthetic enzymes as possible drug targets for antifungal and antiprotozoals. Consequently, inhibitors of many of the steps in sphingolipid biosynthesis have been described [2, 43]. Before the synthesis of ceramide/phytoceramide the sphingolipid biosynthetic pathway is largely conserved across evolution. At least in part because of this, all the inhibitors identified as targeting fungal enzymes in this part of the biosynthetic pathway are nonselective and inhibit the mammalian orthologues [43]. This has curtailed their clinical application as anti-fungal agents, for example fumonisin B_1_ which inhibits the fungal phytoceramide synthase demonstrated mammalian toxicity [93]. In contrast the post-ceramide divergence represented by the absence of IPC synthase and inositol-based sphingolipids in mammalian cells, highlights the therapeutic potential of inhibitors targeting fungal IPC synthases. Such inhibitors could result in selective antifungal drugs with minimal host toxicity. Additionally, the identification and isolation of functional orthologues of the fungal enzyme in the kinetoplastid protozoan parasites (*Leishmania *spp., *Trypanosoma brucei *and *Trypanosoma cruzi*) indicated that IPC synthase is a valid target for antiprotozoal compounds [3]. One of the *T. brucei* sphingolipid synthases, a novel bifunctional enzyme catalysing the synthesis of both IPC and SM, is essential for parasite growth and can be inhibited *in vitro* by the antifungal aureobasidin A at low nanomolar concentrations [43]. As the causative agent of Chagas' disease, *Trypanosoma cruzi* has a complex lifecycle with an essential intracellular stage in vertebrate hosts, in addition to an extracellular existence in an insect vector. Necessary to persistence of the lifecycle is the synthesis of surface glycosylphosphatidylinositol (GPI) anchored glycoconjugates, meaning that the biosynthesis of GPI anchors is attractive target for new therapies against Chagas' disease [93]. As many *T. cruzi* GPI anchors contain IPC as the lipid portion, the sole IPC synthase is highlighted as a new therapeutic target for Chagas disease.

To date only the natural compounds—aureobasidin A [33, 94, 95], Khafrefungin [94], and Rustmicin [96, 97] have been reported as potent inhibitors of the fungal IPC synthase. As discussed, there is an inhibitory effect of aureobasidin A against the Kinetoplastid enzyme orthologues [43, 98, 99], although the specificity of this remains unclear [3, 100, 101]. Unfortunately, further development of all the three known inhibitors of IPC synthase has stalled either due to lack of physical properties required for an acceptable pharmacokinetic profile [42, 102], or because their highly complex structures render chemical synthesis challenging. Moreover, the few synthetic efforts to modify or synthesise analogues that have been reported, resulted in compounds with either reduced or no activity [103, 104]. What has proven more successful however is the development of substrate (ceramide) analogues, with targeted inhibition against the protozoal IPC synthases *in cellulo* [105].

In addition to sphingolipid synthesis as a therapeutic target against parasitic protozoa, degradation of sphingolipids similarly is an area providing new opportunities for antiprotozoal compounds. *Leishmania *spp. use sphingolipid biosynthesis to generate ethanolamine (Etn), essential for the survival and differentiation from procyclics to virulent metacyclics [21]. A likely starting point for Etn production is the degradation of IPC, and a putative neutral SMase and/or IPC hydrolase (IPCase), designated *ISCL* was identified in the *L. major* genome [106]. ISCL showed much greater activity against non-self SM over IPC, suggesting a role in host SL degradation confirmed by *ISCL* null mutants failing to induce lesions in susceptible BALB/c mice. Further investigation revealed that host SL catabolism by *Leishmania* was essential to resist the harsh acidic environment in the phagolysosomes of macrophages [107]. These findings reveal that SL catabolism, as well as anabolism, by *Leishmania* is necessary for proliferation of the parasite in the mammalian host, making the ISCL enzyme an equally attractive target for inhibition studies. *Trypanosoma cruzi* invades mammalian cells by attaching and mimicking injury to the host plasma membrane, inducing a repair process that involves the Ca^2+^-dependent exocytosis of lysosomes [108]. As host acid sphingomyelinase (aSMase) is delivered by lysosomes to the plasma membrane, its ceramide-generating activity promotes rapid endocytosis to internalise the seemingly damaged membrane and the attached parasites. Consequently any inhibition or reduction of this lysosomal aSMase blocks *T. cruzi* invasion, though subsequent treatment with an extracellular sphingomyelinase can restore the infection to normal levels. In a similar approach it has been demonstrated that inhibition of host cell SPT, and so sphingolipid biosynthesis, by myriocin, suppresses hepatitis C virus replication [109]. In addition, it has emerged that ceramide induces activation of double-stranded RNA-dependent protein kinase-mediated antiviral response [110]. These recent findings suggest that manipulation of host sphingolipid metabolism may provide a new combined therapeutic strategy for treatment of both protozoal and viral infections.

## 6. Perspective

Recent studies of the genetic, biochemical and cell biology of sphingolipids have provided exciting new insights into their function, regulation and control, allowing the consideration of future manipulations to aid the fight against human diseases including cancer and major fungal and parasitic infections. In addition, this information may ultimately aid the treatment of several rare genetic disorders (e.g. the sphingolipidoses) and, perhaps, Alzheimer's disease. However, in most cases these studies are at an early stage and further work is required to establish proof of concept. This will undoubtedly be achieved as progress towards a fuller understanding of the complex and multilayered metabolic pathways of sphingolipid metabolism is realized, and as inhibitors of the enzymes involved become available.

## Abbreviations

SM: Sphingomyelin
IPC: Inositol phosphorylceramide
MIPC: Mannose-IPC
PI: Phosphatidylinositol
DAG: Diacylglycerol
CS: Ceramide synthase
CDes: Ceramide desaturase
SMS: Sphingomyelin synthase
SL: Sphingolipid
PC: Phosphatidylcholine
SMases: Sphingomyelinases
aSMase: Acid sphingomyelinase
Alk-SMase: Alkaline SMase
SPT: Serine palmitoyltransferase
3-KSR: 3-Ketosphingosine reductase
CerS: Ceramide synthase
CDase: Ceramidase
CDes: Ceramide desaturase
SMS: Sphingomyelin synthase
SMase: Sphingomyelinase
CK: Ceramide kinase
GCS: Glucosylceramide synthase
GCase: Glycosidases
SK: Sphingosine kinase
SPP: Sphingosine-1-phosphate phosphatase
S1P: Sphingosine-1-phosphate
HSN1: Hereditary sensory neuropathy type I.
